# Correlation between respiratory function and spine and thorax deformity in children with mild scoliosis

**DOI:** 10.1097/MD.0000000000007032

**Published:** 2017-06-02

**Authors:** Andrzej Szopa, Małgorzata Domagalska-Szopa

**Affiliations:** School of Health Sciences in Katowice, Medical University of Silesia, Poland.

**Keywords:** idiopathic scoliosis, Moiré topography, pulmonary function test, scoliosis screening

## Abstract

Idiopathic scoliosis (IS) is the most common 3-dimensional deformation abnormality of the spine with direct effects on the thoracic cage and can potentially affect respiratory function.

The purpose of the present study was to recognize whether the 3-dimensional displacement of the spine and trunk as a consequence of IS directly influences and diminishes respiratory function in children with mild IS.

The study involved 68 children aged 10 to 12 years with mild thoracic or thoracolumbar IS who were the outpatients of the local Center for Corrective Gymnastics. The study consisted of 2 interrelated parts: the body posture examination using a Moiré topography and the spirometric examination including measurements of basic ventilatory parameters (vital capacity [VC], forced vital capacity [FVC], forced expiratory volume in 1 second [FEV_1_], and FEV_1_/FVC).

For the majority of subjects, the results of VC were within the normal range and did not confirm the existence of features characteristic for ventilatory functional restriction. The VC does not depend on the curvature angle value or the degree of rotation of vertebral bodies. It was observed that VC in children with mild IS of 20 to 30 degree depended on thoracic kyphosis, that is, length, depth, and the thoracic kyphosis length/depth indicator.

The results of performed study showed that in children with mild IS the lung volumes are reduced not only by an increased angle of the lateral curvature but also by the degree of loss of normal thoracic kyphosis. The regular respiratory function testing and back-shape analysis are advisable in children with thoracic and thoracolumbar mild IS.

## 1 Introduction

Idiopathic scoliosis (IS) is the most common 3-dimensional deformation abnormality of the spine with an overall prevalence of 0.47% to 5.2% presented in the current literature.^[1–3]^ The female to male ratio ranges from 1.5:1 to 3:1 and increases substantially with increasing age. In particular, the prevalence of curves with higher Cobb angles is substantially higher in girls than in boys.^[1]^

The overweight children are more frequently exposed on development of scoliosis and tend to have larger curves of scoliosis.^[4]^

Although the lateral curvature dominates in IS, this common disorder of 3-dimensional deformity of the spine and trunk has many features that can negatively affect respiratory function, including the lateral displacement and rotation of vertebral bodies, sagittal thoracic kyphosis, and lumbar lordosis during somatic growth. These primary impairments owing to IS can lead to secondary impairments, such as decreasing the chest wall and reducing lung compliance, resulting in increased effort to breathe at rest and/or during physical activity, especially in children with severe IS.^[5–9]^

Among children with IS large intersubject differences are observed both in clinical images as well as in the natural history of scoliosis. One of the primary reasons for this is the considerable diversity of individual compensatory mechanisms. Numerous studies on the function of the respiratory system during the course of scoliosis were “correlated” primarily with the value of the Cobb angle, and their main goal was to determine the limiting Cobb angle after which impairment of respiratory system function occurred.^[10–15]^ Data from the literature indicate that study results in this respect are ambiguous and may even be contradictory. Some authors suggest that respiratory defects occur in scoliosis cases in which the Cobb angle exceeded 50 or 65 degrees.^[10–13]^ Meanwhile, others have already confirmed the above defects in mild scoliosis, that is, where the scoliosis angle does not exceed 30 degree.^[14,15]^ Most of the above-cited studies analyzed various respiratory system parameters only with regards to the Cobb angle value; these studies did not examine other equally important scoliosis features such as the lateral displacement and rotation of vertebral bodies and sagittal thoracic kyphosis.

Moiré topography (MT) is an imaging method for the body surface and is highly sensitive in the detection of asymmetry.^[16–20]^ Historically, MT was based on the interference of grids projected onto the subject's back^[21]^; the current methods are based on computerized image captured and digitally calculated parameters. A few studies have reported a high correlation between Moiré angle analysis and radiographic analysis of the spinal curvature.^[16–18]^

In the present study, the analysis of the angle of the spine's lateral curvature while taking into account other 3-dimensional deformities of the spine and trunk based on MT examination were combined with spirometry testing and have created new possibilities for the functional evaluation in children with IS. Angle of the vertebral lateral curvature (ALC) is related to the Cobb angle definition, but it is not the same value as the Cobb Angle. Because the Cobb angle can be obtained only with x-ray measurements, back surface indices were invented to simulate the Cobb angle; however, a few studies reported a high correlation between Moiré angle analysis and radiographic analysis of the spinal curvature.^[16,18,22]^

The purpose of the present study was to recognize whether the 3-dimensional displacement of the spine and trunk as a consequence of IS directly influences and diminishes respiratory function in children with mild IS. We hypothesized that diminishing respiratory function developed in children with mild scoliosis was not dependent only on the angle of the spine's lateral curvature, but on the secondary scoliotic deformity referred to as thoracic cage deformity.

## 2 Material and methods

The research protocol was approved by the Local Bioethical Committee (KNW/0022/KB1/89/15) and parents/guardians provided signed, informed consent before the subjects’ enrollment in the study.

### 2.1 Study design: cross-sectional observational study

From all of 93 children with a diagnosis of IS, who were the outpatients of the local Center for Corrective Gymnastics from January 1, to December 31, 2015, only 74 met the inclusion criteria for the study. However, of 74 eligible, identified individuals, a representative sample of 68 participated in the study. The study involved 54 girls and 14 boys, aged 10 to 12 years with mild idiopathic thoracic scoliosis (range of major curvature 5–30 degree), who were treated conservatively using physiotherapy based on the Schroth scoliosis Exercise method (PH) or PH with Night Time Rigid Bracing (12–20 hours per day) (Table 1).

**Table 1:**
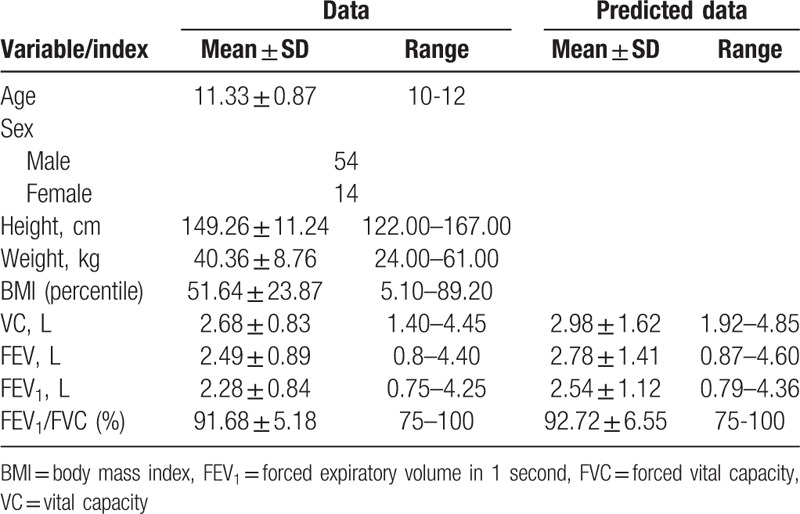
Baseline characteristics of study group.

They were diagnosed by a physician as having IS, based on the clinical criteria of IS:

- unknown etiology of scoliosis
- level of major curve: thoracic
- developmental and 3-dimensional torsional deformity of the spine
- major lateral curve angle >5 degree with monitored progression during growth.

All subjects met the following inclusion criteria: older than 7 years of age, able to follow verbal directions, mild thoracic or thoracolumbar scoliosis (level of major curve: thoracic) (ALC <30 degree), no previous surgical procedures. The exclusion criteria included: thoracic surgery and obstructive ventilatory defects, the latter determined by the value of forced expiratory volume in the first second (FEV_1_) in relation to vital capacity (VC), wherein a FEV_1_/forced vital capacity (FVC) ratio <70% led to study exclusion, previous respiratory complaints.

The study consisted of a 2 interrelated parts:

(1) the MT examination using a CQ Electronic System (Poland)
(2) the spirometric examination including basal ventilatory parameter (VC, FVC, FEV_1_, and FEV_1_/FVC) measurements using a Micro Lab MK8 Viasys spirometer.

### 2.2 MT examination

For the MT examination, it was necessary to uncover the entire surface of the back and to mark some anatomical landmarks. These landmarks were the spinous processus from C7 to S1, the acromial angle of the shoulders, the superior angle of the scapula, the inferior angle of the scapula, and the posterior superior iliac spine, as suggested by the Society on Scoliosis Orthopedic and Rehabilitation Treatment.^[23]^

**Figure 1:**
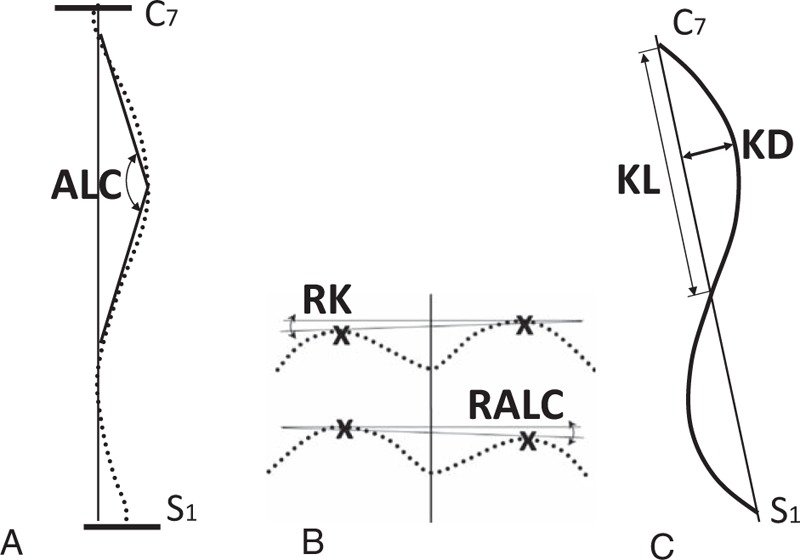
Moiré topography indices. (A) Indices measured on the coronal plane:angle of the major vertebral lateral curvature (ALC) (degree); (B) indices measured on the transverse plane: angle of spinal rotation at the level of the apex of kyphosis (RK) (degree); angle of spinal rotation at the level of the apex of the major curvature (RALC) (degree); (C) indices measured on the sagittal plane: the length of the kyphosis (KL) (millimeters); the magnitude of the maximum kyphosis − depth – (KD) (millimeters).

In the literature, many indices are computed in each of the 3 planes. For the purposes of the present study, the following indices were chosen:

(1) ALC (degree) measured on the coronal plane, related to the Cobb angle (Fig. 1A) definition. ALC have values that range from 180 to 90 degree, and the angle of scoliosis is larger when the ALC is close to 90 degree.
(2) Indices measured on the sagittal plane refer to the magnitude of the maximum kyphosis (Fig. 1C):
  (a) the length of the kyphosis (KL) (millimeters)
  (b) the magnitude of the maximum kyphosis – depth (KD) (millimeters)
  (c) thoracic kyphosis length/depth indicator (KI) (KI = KL/KD)
(3) Indices measured on the transverse plane (Fig. 1B):
  (a) the angle of spinal rotation at the level of the apex of kyphosis (RK) (degrees).
  (b) the angle of spinal rotation at the level of the apex of the major curvature (RALC) (degrees)

The angle of surface rotation (α angle) was contained between 2 adjacent lines: a line situated within the frontal plane and a line that connected 2 points on the back surface and was situated symmetrically on the left and right sides of the corresponding spinous process at the level of the RK and RALC.

The MT examination was performed by 1 observer using a CQ Electronic System (Poland). Two experienced physical therapists selected both the Moiré photographs and the measurement of body weight distribution. The image that was the most characteristic of the child was chosen for further analysis. When the 2 experts agreed, the arithmetic mean of their assessments was recorded. When their assessments differed, the senior author (MDS) chose the image that was analyzed. The accuracy of their evaluations was then analyzed. The repeatability of MT examination was assessed based on the value of intraobserver error and interobserver error as outlined in the study by Chowańska et al. They stated that CQ surface topography evaluation has a good repeatability and reproducibility.^[16]^

### 2.3 Spirometry

VC and FVC were tested in a standing position using a Micro Lab MK8 Viasys automatic spirometer. Both measurements were taken 3 times and averaged. The vital capacity indicator (VC%) was used for further analysis. FVC and FEV_1_ and the FEV_1_/FVC ratio were used only for the exclusion criteria.

### 2.4 Statistical analysis

Data were analyzed using the Statistica Version 12.0 PL statistical software program. The intraobserver and interobserver MT measurements were assessed. The intraclass correlation coefficient (ICC) with a 95% confidence interval was used. Intraobserver and interobserver agreement was calculated for each MT index based on 2 examinations performed by the same 2 researchers of 10 subjects (40 examinations in total). Interobserver agreement was calculated (for the same subjects) for 2 of the reviewers (ICC 3.2). For the analysis, a mean ICC value of ≥0.80 indicated excellent reliability, those between 0.70 and 0.79 indicated good reliability, and those <0.70 indicated poor-to-moderate reliability.^[24]^ Descriptive statistics for the data of scoliosis deformity indices and the VC% are expressed as the mean and standard deviation. Normality for both was verified by the Kolmogorov-Smirnov test. The dependence of scoliosis deformity indices on the VC% was analyzed based on the Pearson correlation coefficient. Coefficients with a *P* value of .05 were considered as significant. The correlations were interpreted according to the guidelines adopted from Altman,^[25]^ wherein *r* <0.20, poor; 0.21 to 0.40, fair; 0.41 to 0.60, moderate; 0.61 to 0.80, good; 0.81 to 1.00, very good.

## 3 Results

The mean VC% was 91.48 ± 22.67 and oscillated within the range of 48% to 163%. The distribution of the VC% values in the study group was as follows: 25% of patients; lung VC below due value (VC% <75%), 38% of patients; lung VC within due norm (75% < VC% >100%), 37% of patients; lung VC above due value (VC% >100%).

Every indicator from the MT examination demonstrated from good to very high level of intraobserver agreement, with the ICC values ranging from 0.75 to 0.98. The ICC values indicated a very high level of interobserver agreement among researchers, with the ICC values ranging from 0.90 to 0.99.

Owing to the large differentiation of individual ALC values, the subjects were divided into 3 clinical groups according to ALC values: A1, ALC 5to 10 degree (33%); A2, ALC 11 to 20 degree (33%); and A3, ALC 21 to 30 degree (34%). The descriptive statistics of MT indices are presented in Table 2.

**Table 2:**
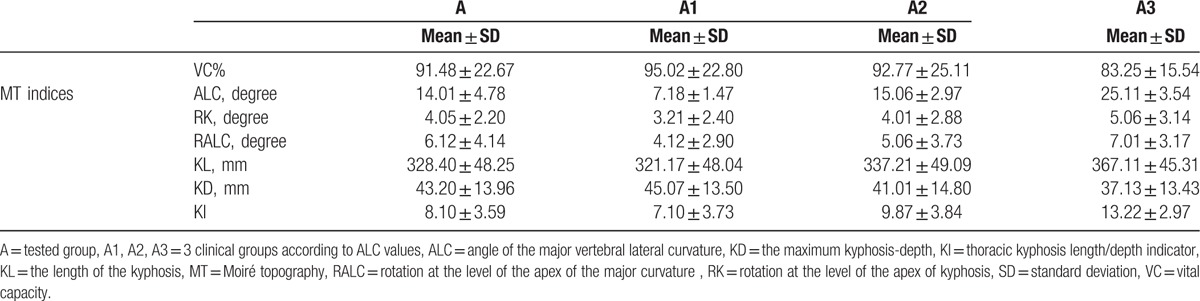
MT indices and the VC% in children with mild idiopathic thoracic scoliosis.

The correlation coefficients between 3 scoliosis deformity indices (ALC, RK, and RALC) as well as between 3 kyphosis deformity indices (KL, KD, and KI) and the VC% are summarized in Table 2. None of the correlations were good or very good. The statistically significant correlations printed in Table 3 were fair (0.21–0.40) to moderate (0.41–0.60) (Table 3).

**Table 3:**
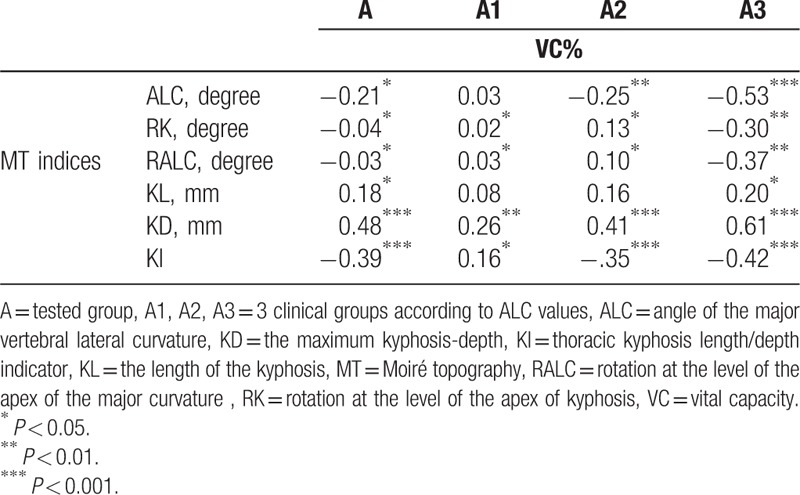
Correlations between the MT indices and the VC% in children with mild idiopathic thoracic scoliosis.

Across the entire tested group, no significant correlations between the vital capacity and scoliosis deformity indices, that is, ALC, RK, and RALC, were found. In general, across the entire tested group, the significant correlations concerned only the thoracic kyphosis deformity parameters, that is, KD and KI (Table 3).

The division of subjects into clinical subgroups taking into account the severity of scoliosis (A1, A2, and A3) revealed the significant correlations of the vital capacity with the scoliosis deformity indices in children with the highest values of the spine's lateral curvature (group A3). Two fair negative correlations between vital capacity and ALC, and rotation at the level of the apex of the lateral curvature were noted (Rs = −0.53, *P* < .001 and Rs = −0.37, *P* < .01, respectively).

## 4 Discussion

The main purpose of the present research was to inspect whether and to what extent spinal deformation impacts the function of the respiratory system in children with mild scoliosis. In light of the obtained results, the answer to this question is not unambiguous. The present study revealed that for the majority of patients with IS (75%), the values of the basic ventilation indicator, that is, the percentage of due lung VC%, were within the normal range and did not confirm the existence of features characteristic for restrictive ventilatory failure, as reported in a few studies.^[16,17]^ A general comparison of all correlation tables revealed that the primary morphological feature of scoliosis, that is, the thoracic vertebral lateral curvature angle value and the degree of rotation in 2 levels, had no significant correlation with the basic functional parameters of the respiratory system in children with mild scoliosis. Moreover, while analyzing the secondary features of scoliosis related to the impact of thorax deformity on lung VC, it was observed that it depends on the KD as well as on the KL/KI. The calculated correlation coefficients showed that lung VC decreased along with the reduction in the curvature of kyphosis, which usually accompanies the progression of scoliosis.

On the contrary, the additional division of patients into clinical groups taking into account the severity of scoliosis (A1, A2, and A3) revealed that in children with the highest angles of scoliosis (20–30 degree), VC depended on primary scoliotic deformity indices, that is, the lateral curvature angle value and the degree of rotation at both levels (apex of kyphosis and apex of the major curvature) and decreased together with an increase in either parameter.

The above observations seem to be confirmed by the results of some studies, which reported a significant and strong positive correlation between VC and Cobb angle in scoliosis exceeding 20 degree.^[9,22,26]^ Taking into account the findings of Lonstein and Carlson^[22]^ as well as those by Nachemson et al,^[26]^ which stated that the largest percentage of scoliosis progression in children occurred with a curvature angle between 20 and 30 degree and with a simultaneous Risser test result of 0 and 1, or within the ages of 10 to 12 years, were entirely coherent with the results obtained in the present study.

Whereas the obtained results indicated that in children with angle of scoliosis between 10 and 20 degree, the features which clearly impacted the lung VC were not primary scoliosis features such as vertebral lateral displacement or spinal rotation, but the secondary scoliosis feature, that is, the thoracic kyphosis, was determined by its depth as well as the KL/KI.

The revealed in present study dependence of the kyphosis profile in the sagittal (A-P) plane on the lung VC has been suggested that tendency to flattening of kyphosis represented by reduction of kyphosis depth, combined with lung VC reduction, could be a risk factor for scoliosis progression among the children with mild IS. This phenomenon was confirmed by studies performed by Winter et al (1992) and Grivas et al (2002). Tsiligiannis et al (2012) and Kotwicki (2002) stated that normal thoracic kyphosis as a backward curvature in an anteroposterior (medial) plane provides physiological mobility of the spine, guarantees its proper statics and properly distributes the body center of gravity in the sagittal plane, thus protecting the spine against deformity.^[9,27–29]^

This fully justifies the author's intentions to extend the presented research to a large population of children with heightened risk of scoliosis progression, that is, children aged 10 to 12 years with mild scoliosis (within the thorax scoliosis range of 5–30 degree) with tendency toward reduced thorax kyphosis and a lung VC below due value (VC% <75%).

The present study showed that MT is a highly repeatable method for the evaluation of the primary and secondary features of thoracic and thoracolumbar mild scoliosis. Utilizing MT for body posture analysis provides reliable quantitative information not only about the ALC but also with regards to trunk deformities. A high sensitivity of MT examination and the diagnostic accuracy for the early detection of scoliosis was reported by Karachalios et al (1999)^[19]^ and Chowańska et al.^[16]^ Evaluation of scoliosis risk progression by means of MT may decrease the number of radiographs obtained during the follow-up of scoliosis.^[1,3,19,20]^

Thus, the results of studies on the function of the respiratory system in patients with scoliosis presented in this work are of extensive practical value. Recreating physiological thoracic kyphosis should be the basic stage of treating IS—conservative, corset, or surgical treatment. The 3-plane correction of scoliosis is achieved only when physiological thoracic kyphosis is recreated. So-called corrective exercises, involving the strengthening of the dorsal muscles operating on the bowstring of the scoliosis arch and the bowstring of the lordosis arch, may unnecessarily lead to lordozation of the thoracic spine and increase the progression of scoliosis.

The number of participants in the study as well as the fact that included population was a convenience sample (children with mild IS) and not based on any specific sample size calculation constituted an important limitation of present study. However, considering the number of examined participants, we are sure these findings may be considered preliminary findings and further studies with a larger number of participants taking into account different degree of severity of IS (moderate, severe, and very severe) are needed.

## 5 Conclusions

In conclusion, the results presented in this work are not meant to negate the existence of defects in the function of the respiratory system in the case of IS, but merely provide some evidence that undermines the unambiguity of the presented problem. The relationship between pulmonary impairment and the spine and thorax deformity in the course of scoliosis is complex and not only depend on curve progression. As shown in the present study, lung volumes are reduced not only by an increased ALC but also by the degree of loss of normal thoracic kyphosis. Because the pulmonary disorders may not be clinically evident until irreversible changes in lung function have already occurred, early recognition of this problem by regular respiratory function testing and body posture examination using back-shape analysis methods are advisable in children with thoracic and thoracolumbar mild scoliosis.
